# Whole Genome Profiling provides a robust framework for physical mapping and sequencing in the highly complex and repetitive wheat genome

**DOI:** 10.1186/1471-2164-13-47

**Published:** 2012-01-30

**Authors:** Romain Philippe, Frédéric Choulet, Etienne Paux, Jan van Oeveren, Jifeng Tang, Alexander HJ Wittenberg, Antoine Janssen, Michiel JT van Eijk, Keith Stormo, Adriana Alberti, Patrick Wincker, Eduard Akhunov, Edwin van der Vossen, Catherine Feuillet

**Affiliations:** 1 INRA-UBP, UMR1095, Genetics Diversity and Ecophysiology of Cereals, 234 Avenue du Brezet, 63100 Clermont- Ferrand, France; 2 Keygene N.V., Agro Business Park 90, 6708 PW, Wageningen, The Netherlands; 3 Amplicon Express Inc., 2345 NE Hopkins Ct., Pullman, WA, USA; 4 CEA-CNS, 2 rue Gaston Crémieux, CP5706, 91057 Evry cedex, France; 5 Department of Plant Pathology, Kansas State University, Manhattan, KS, USA

## Abstract

**Background:**

Sequencing projects using a clone-by-clone approach require the availability of a robust physical map. The SNaPshot technology, based on pair-wise comparisons of restriction fragments sizes, has been used recently to build the first physical map of a wheat chromosome and to complete the maize physical map. However, restriction fragments sizes shared randomly between two non-overlapping BACs often lead to chimerical contigs and mis-assembled BACs in such large and repetitive genomes. Whole Genome Profiling (WGP™) was developed recently as a new sequence-based physical mapping technology and has the potential to limit this problem.

**Results:**

A subset of the wheat 3B chromosome BAC library covering 230 Mb was used to establish a WGP physical map and to compare it to a map obtained with the SNaPshot technology. We first adapted the WGP-based assembly methodology to cope with the complexity of the wheat genome. Then, the results showed that the WGP map covers the same length than the SNaPshot map but with 30% less contigs and, more importantly with 3.5 times less mis-assembled BACs. Finally, we evaluated the benefit of integrating WGP tags in different sequence assemblies obtained after Roche/454 sequencing of BAC pools. We showed that while WGP tag integration improves assemblies performed with unpaired reads and with paired-end reads at low coverage, it does not significantly improve sequence assemblies performed at high coverage (25x) with paired-end reads.

**Conclusions:**

Our results demonstrate that, with a suitable assembly methodology, WGP builds more robust physical maps than the SNaPshot technology in wheat and that WGP can be adapted to any genome. Moreover, WGP tag integration in sequence assemblies improves low quality assembly. However, to achieve a high quality draft sequence assembly, a sequencing depth of 25x paired-end reads is required, at which point WGP tag integration does not provide additional scaffolding value. Finally, we suggest that WGP tags can support the efficient sequencing of BAC pools by enabling reliable assignment of sequence scaffolds to their BAC of origin, a feature that is of great interest when using BAC pooling strategies to reduce the cost of sequencing large genomes.

## Background

With Next Generation Sequencing (NGS) technologies, sequencing costs have been reduced by over 10,000 times during the past ten years [1] thereby opening new opportunities for genome sequencing of model plants and crops [2]. The sequencing strategy and the quality of the genome sequence assembly depend on the objectives of the analysis and the complexity of the target genome (size, repeat content) [2,3]. Whole Genome Shotgun (WGS) and clone-by-clone strategies have been used separately or in combination to obtain plant genome sequences [4]. The main advantage of a clone-based approach compared to WGS is that it facilitates the accurate assembly of regions harboring multigene families and transposable elements (TEs) that are repeated at the genome level but not at the clone level [5]. Such sequences collapse into single contigs in WGS assemblies, particularly when data are obtained using the NGS short read technologies, and therefore biologically relevant information is lost. Moreover, the physical maps that are constructed for the clone-based approach enable direct links between the sequence and genetic maps carrying genes and quantitative trait loci (QTL) of interest thereby enhancing the value of the genome sequence for breeders. For these reasons, the clone-by-clone approach remains a gold standard for sequencing species that contain a high level of repetitive elements and are of economic interest such as maize and wheat (~85% of TEs) [6].

Sequencing projects using a clone-by-clone approach require the availability of a robust physical map, *i.e*. an ordered list of contiguous overlapping large inserts cloned into a vector (commonly, a bacterial artificial chromosome (BAC)). Different methodologies including hybridization, Sequenced Tag Site mapping, and fingerprinting with restriction enzymes [7-17] have been employed to build physical maps. Fingerprinting has been commonly used in the past decades to build physical maps of animal and plant reference genomes (e.g. human, *Arabidopsis thaliana *and rice [11,18,19]. Briefly, it consists of digesting BAC clones with one or more restriction enzymes [7,10,12,13] and performing pair-wise comparisons of the restriction profiles to build contigs based on the assessment of overlaps between the fingerprints [20]. Numerous techniques using different types of restriction enzymes, different migration support, and/or different size calling methods have been developed [10,13,21,22]. A comparative study between the five main technologies concluded that the five-enzyme method called SNaPshot is the most effective to build physical maps [10,12]. This method has been used recently to build the first physical map of a wheat chromosome (chromosome 3B, 1 Gb [23]) and to complete the maize physical map [24].

A high quality physical map should meet several criteria. First, the number of contigs representing a chromosome or a genome should be as low as possible to support contig ordering and orientation and subsequently reduce the sequencing costs. The physical contigs should also contain a minimal percentage of mis-assembled BACs (*i.e*. BACs comprised in a physical contig which do not correspond to the genomic region covered by the contig) and a minimal percentage of chimerical contigs (*i.e*. contigs covering non-contiguous regions). The latter two criteria are even more important for physical mapping in complex genomes that have a high level of repeats (> 80%) as such sequences tend to increase the number of bands shared randomly between two non-overlapping BACs, thereby increasing the percentage of mis-assembled BACs and chimerical contigs. In the maize physical map, the average number of shared bands between two random BACs was 10.8 from an average 98 bands per BAC, i.e. an average random overlap of 11% [25]. For the wheat 3B chromosome physical map developed with the SNaPshot technology, the average random overlap of bands was ~10% [23].

Whole Genome Profiling (WGP™) has been developed recently as a new sequence-based physical mapping technology [26]. It first consists of pooling an arrayed BAC library with pooling complexities and dimensions that are adapted to the genome size. Then, pools of BAC DNA are digested, the restriction fragments are ligated with adaptors, and the adaptor-ligated fragments sequenced using the Illumina Genome Analyzer (GA) II sequencer to yield restriction fragment (WGP) tags of 26 to 31 nt. Deconvolution enables the assignment of these sequence tags to individual BACs. Pair-wise comparisons of tags allow identifying BAC overlaps and assembling contigs using the FingerPrinted Contigs (FPC) software [20,27]. The requirement for perfect sequence matches between tags in WGP assemblies allows for a stringent cut-off setting in FPC and thus the construction of robust physical maps. The WGP technology has been applied recently in *A. thaliana *using a 6x BAC library, resulting in 357 contigs that covered 102 Mb or 80% of the *Arabidopsis *genome with 97% of the contig coverage validated by mapping tags to the reference sequence [26].

Here, we evaluated the potential of WGP for physical mapping in wheat as a proof of concept for the International Wheat Genome Sequencing Consortium (IWGSC) that is currently establishing a physical map of the 21 chromosomes of bread wheat http://www.wheatgenome.org. We used a subset of 27% of the wheat 3B chromosome BAC library [23] representing 230 Mb to establish a WGP physical map and compare it with a SNaPshot map constructed using the same BACs. The results show that with an improved assembly methodology WGP resulted in 30% fewer contigs than SNaPshot and that the WGP contigs contained 3.5 times less mis-assembled BACs. Finally, we performed a series of chromosome 3B BAC contig sequence assemblies using different types of Roche 454 reads at different coverage levels to assess the potential of WGP tag integration for supporting wheat genome sequencing. The results showed that while the integration of WGP sequence tags improves low quality assemblies, it does not reduce significantly the level of sequencing coverage (and cost) necessary to achieve a high quality reference sequence because of the inherent complexity of the wheat genome and the limited number of deconvoluted tags per BAC with the current approach. Nevertheless, those tags are extremely useful for assigning sequence scaffolds to their BAC of origin in sequencing projects where several BACs are pooled.

## Results

To compare the efficiency of the Whole Genome Profiling and SNaPshot technologies for establishing sequence-ready physical maps in the 17 Gb wheat genome, we performed an experiment on a subset of about one third of the 9x BAC library of chromosome 3B that was used to build the chromosome 3B physical map with the SNaPshot technology [23]. In this map, 56,952 fingerprints were assembled into 1991 contigs (including 44,008 BACs) and 12,944 singletons that cover 811 Mb. Here, we randomly picked ~27% of the singletons (3623 BACs) and ~27% of the 1991 contigs (527 contigs including 11,125 BACs) to perform physical mapping. In addition, we included 1380 BACs that originated from 12 Mb- sized contigs (17.9 Mb) recently sequenced and fully annotated [28]. These were used as references to assess the accuracy of the physical map assemblies. All in all, the subset used for comparing WGP and SNaPshot physical mapping results contained 16,128 BACs representing 230 Mb with 9.6X coverage.

### Whole Genome Profiling of 230 Mb of wheat chromosome 3B

The selected 16,128 wheat BACs were subjected to WGP primarily following the method described by Van Oeveren *et al *[26]. Briefly, the BACs were pooled from 42 384-well plates (i.e. 384 BACs with a 138 kb average insert size which per plate covers ~53 Mb (23%) of the 230 Mb), in a 3-D fashion into row, column and splitbox pools resulting in 22 pools per plate (924 pools in total). After digestion of the DNA pools by *Eco*RI and *Mse*I and ligation of barcoded adaptors, amplified fragments were sequenced from the *Eco*RI restriction site ends using the Illumina GAII sequencer. In total, 101.2 million passed filter reads with 36 nt read length were obtained [EMBL:ERP000818]. Of these reads, 95% (96.3 million) carried a valid (100% matching) sample identification tag (barcode) and *Eco*RI restriction site sequence. It was possible to deconvolute 42% of the sequence tags (*i.e*. assign them to a single BAC without ambiguity). In total, 327,282 tags were generated representing 111,678 non-redundant tag sequences that were assigned to 14,199 BACs (Table 1). The 1,929 BACs without any assigned tags likely result from small insert or "empty" BACs, technical problems during pooling and sequencing and/or occasional presence of overlapping BACs from the same region. A filtering step was applied to eliminate tags matching vector, *E. coli*, or chloroplast sequences, tags containing homopolymer sequences of five nt or longer, tags considered uninformative, *i.e*., present in only one BAC (17.3%), and tags potentially introducing ambiguities, *i.e*., present in more than 12 BACs (0.8%). This reduced the number of tags to 47,900 non-redundant tags (228,282 in total) assigned to 13,888 BACs.

**Table 1:** Characteristics of the sequence tags produced by WGP

	Expected data	Raw data	Tag filtering	BAC filtering
Total number of tags	5,681,912	327,282	228,263	194,716
Total number of unique tags	97,293^a^	111,678	47,900	47,220
Average tag length (bp)	30.0	30.0	29.9	29.9
Number of BAC with tag	16,128	14,199	13,888	11,238
Average number of tag per BAC	58.4^b^	23.0	16.4	17.3
Average number of BAC sharing the same tag	9.6	2.9	4.8	4.1

^a ^based on a average *Eco*RI sites density of 4,728 bp and a coverage of the BACs subset of 230 Mb.

^b ^based on a average *Eco*RI sites density of 4,728 bp and an average BAC size of 138 Kb.

#### Impact of the transposable element content on tag distribution

**Figure 1:**
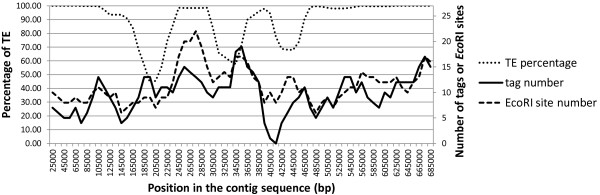
**Tag number, *Eco*RI site number, and TE percentage distribution along a reference contig sequence (ctg1035)**. A window of 50 kb sliding every 10 kb was used to calculate the number of tags, *Eco*RI sites, and the percentage of transposable elements (TE). Each position on the graph corresponds to the middle of each window.

Before building the WGP physical map, we analyzed the distribution of WGP tags along the BACs and investigated whether there is a relationship between the tag distribution and the repeated elements that represent more than 80% of the wheat genome [6]. The number of tags and *Eco*RI sites as well as the TE percentage distribution were computed using sliding windows of 50 kb with steps of 10 kb in the 1380 BACs corresponding to the 12 sequenced contigs [28] used as controls in this experiment. A correlation analysis was performed with a total of 3,396 tags and 3,796 *Eco*RI sites. A representative example is shown in Figure 1. The average distance between two flanking tags was 5,251 bp with a standard deviation (SD) of 7,541 bp (Table 2), while the median was 2,329 bp. In one third of the cases, the distance between two tags was null because tags originated from the same *Eco*RI restriction site. No large regions without tags were observed in the 12 sequences and the maximum distance observed was 84,687 bp, *i.e*. less than the average BAC size (Table 2). The correlation coefficient between the number of tags per BAC and the percentage of TEs was -0.10 (r^2 ^= 0.01, p-value = 0.00003) indicating that the abundance of transposable elements does not impact the distribution of sequence tags, *i.e*. WGP is a resilient technology that can be applied to genomes with high TE content.

**Table 2:** Features of the WGP tag distribution along 18 Mbp of sequence corresponding to 12 reference contigs of chromosome 3B.

Total number of unique tags	3,396
Maximum distance^a ^between two unique tags (bp)	84,687
Minimum distance^a ^between two unique tags (bp)	0
Average distance^a ^between two unique tags (bp)	5,251
Standard deviation^a ^distance between two unique tags (bp)	7,541
Median distance^a ^between two unique tags (bp)	2,329
Percentage of *Eco*RI sites without tags	35.7%
Percentage of sites with two unique tags	38.9%

^a ^distances are calculated between the end of a tag and the beginning of the next one.

#### WGP physical mapping with FPC using parameters used for the construction of the Arabidopsis WGP map

We first used the parameters described by Van Oeveren *et al *[26], *i.e*. a single cut-off at 1e^-06 ^and one DQing step at 1e^-06 ^to perform the assembly. This resulted in 2886 singletons and 786 contigs of which 36% (5.6 chimerical contigs for 10 Mb) were estimated as chimerical and 27% as mis-assembled based on the comparison with the 12 reference contigs (See Materials and Methods). This indicated that the default FPC parameters and/or data filtering were not stringent enough to build a robust physical map for this more complex dataset.

To increase the stringency of the analysis, we performed a second tag filtering with the parameters used previously for assembling the 3B SNaPshot physical map [23]. It consisted in the elimination of BAC fingerprints comprising less than 30% of the average number of tags per BAC (≤ 4 tags; considered as low quality fingerprints) and those with more than 2.5 times the average number of tags per BAC (≥ 40 tags; which is expected to include the vast majority of those that result from chimerical BACs or cross contamination of BACs in the same well, if present). A final set of 47,220 tags assigned to 11,238 BACs, representing only 48.5% of the expected single tags (47,220 vs 97,293) and 29.6% of the expected number of tags per BAC (17.3 vs 58.4), was obtained (Table 1) and processed. Using this approach, the deconvolution and filtering of the WGP data resulted in a loss of 30.4% of the library clones; whereas, only half of them, 16.2%, were discarded using the same criteria during the SNaPshot assembly [23]. The 47,220 tags assigned to 11,238 BACs were then used to build a second version of the physical map using the same FPC parameters described above. The assembly comprised 853 contigs and 1214 singletons. Comparison with the 12 sequenced reference contigs showed that 15.6% of the WGP contigs were chimerical (2.8 chimerical for 10 Mb) and 14.9% of the BACs were mis-assembled. Thus, although the second filtering step improved the quality of the physical map, the amount of chimerical and misassembled contigs was still too high to ensure the construction of a robust physical map, indicating that the FPC parameters used for the construction of *Arabidopsis *WGP maps by Van Oeveren *et al *[26] were not stringent enough for these wheat data.

#### Optimizing FPC assembly parameters for the construction of a robust WGP physical map

**Figure 2:**
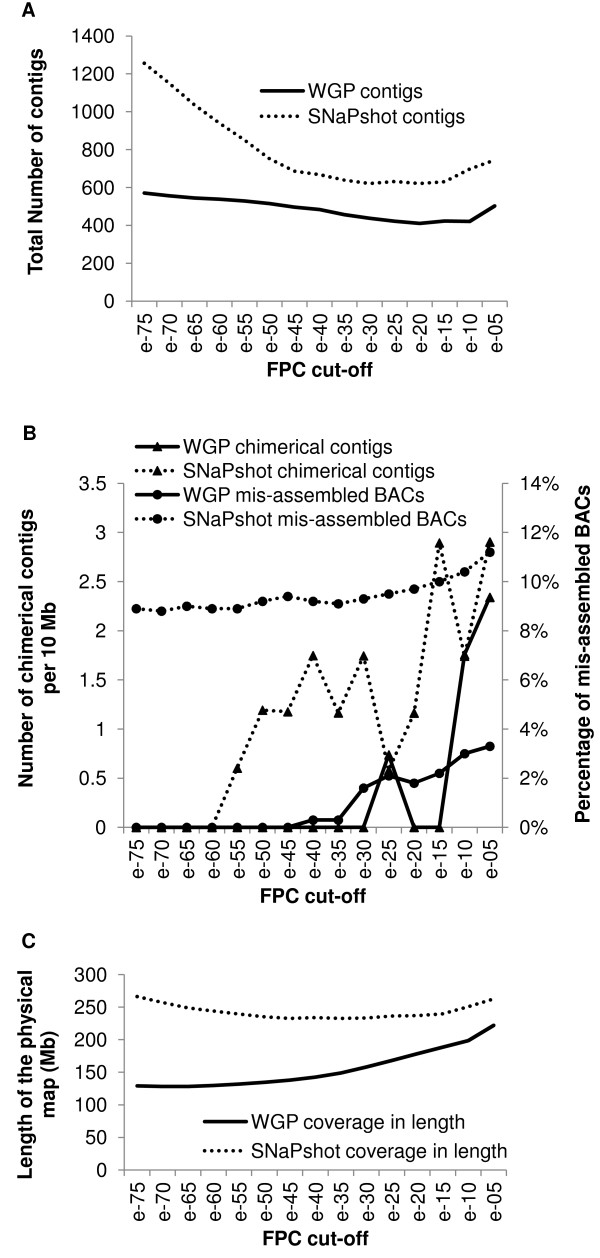
**Analysis of SNaPshot and WGP assemblies built between cut-offs of 1e^-75 ^and 1e^-05^**. A) Total number of contigs at each cut-off. B) Estimated number of chimerical contigs for 10 Mb and percentage of mis-assembled BACs. C) Coverage in length of the WGP and SNaPshot physical assemblies at different cut-offs. The coverage and contigs size were estimated on the basis of an average BAC size of 138 kb and an average band size of 1.1 kb for SNaPshot and 6.1 kb for WGP.

To further improve the robustness of the assembly, we used the stepwise method defined by Paux *et al *[23] for the construction of the 3B chromosome physical map. The method consists of a series of automated FPC assemblies with decreasing stringencies starting with a high cut-off value of 1e^-75 ^and ending at a cut-off of 1e^-45^. Because WGP allows for a tolerance of 0 and has a higher resolution, the final cut-off value can be lower than the one used with the SNaPshot technology (tolerance of 4). Thus, here, we performed 15 assembly steps until a final cut-off of 1e^-05 ^(compared to 1e^-45 ^in Paux *et al *[23]). The initial assembly at 1e^-75 ^resulted in 571 contigs (Figure 2A). Subsequently, the number of contigs decreased until a cut-off of 1e^-25 ^(422 contigs), remained stable until 1e^-10 ^(421 contigs), and increased again at 1e^-05 ^(503 contigs). At the last cut-off, we observed a significant increase of the number of questionable (Q) clones before the DQing steps, part of these Q clones were eliminated by FPC resulting in the split of contigs and consequently in an increased number of contigs (Figure 2A). This showed that for WGP in wheat, reliable assemblies cannot be obtained at cut-offs lower than 1e^-10^. No mis-assembled BAC was detected between cut-offs of 1e^-75 ^and 1e^-45 ^(Figure 2B), while the percentage of mis-assembled BACs increased to 3.3% at a cut-off of 1e^-05^. Up to a cut-off of 1e^-15^, the number of chimerical contig was negligible. At cut-offs of 1e^-10 ^and 1e^-05^, the number of chimerical contigs detected for 10 Mb was 1.8 and 2.3, respectively. We also examined the physical map length at the different cut-offs (Figure 2C). To estimate the map length, we first needed to determine the average size of a CB unit (see materiel and methods). On average, one CB unit corresponds to 6.1 kb ± 1.3 in the WGP maps. The results show that the map length varied from 128 Mb to 222 Mb with the longest map obtained at a cut-off of 1e^-05 ^(Figure 2C).

All together these results show that the optimal window of cut-offs that produces the best physical map assembly, *i.e*. an assembly with (1) a minimum number of contigs, (2) a maximum coverage in length of the physical map, (3) a minimum number of chimerical contigs, and, (4) a minimum percentage of mis-assembled BACs, is between 1e^-15 ^and 1e^-10^. To determine a single optimal value, we performed new assemblies at 1e^-15^, 1e^-14^, 1e^-13^, 1e^-12^, 1e^-11^, and 1e^-10 ^(data not shown). The results indicate that the best value is 1e^-11^. Thus, we conclude that for these wheat data, a robust physical map can be obtained by WGP when using a stringent stepwise, cut-off approach starting at 1e^-75 ^and ending with a final cut-off of 1e^-11^.

### Comparison of SNaPshot and WGP automated physical map assemblies

To compare the results of the WGP and SNaPshot methodologies, we performed a stepwise assembly (initial cut-off of 1e^-75^, 15 steps until a final cut-off of 1e^-05^) of the SNaPshot fingerprints from the 11,238 BACs clones used in WGP (see above). The initial assembly resulted in 1,256 contigs (Figure 2A) and, subsequently, the number of contigs decreased steadily until a cut-off of 1e^-30 ^(621 contigs). From 1e^-25 ^to 1e^-05 ^the number of contigs remained stable and even increased again after 1e^-15 ^to eventually reach 745 at a cut-off of 1e^-05 ^(Figure 2A). This indicates that reliable results cannot be obtained with SNaPshot data in wheat at cut-offs lower than 1e^-15 ^because of the presence of too many Q clones. A comparison with the 12 sequenced contigs enabled us to estimate the number of chimerical contigs and the percentage of mis-assembled BACs at the different steps of the SNaPshot assembly (Figure 2B). The percentage of mis-assembled BACs was relatively stable at cut-offs between 1e^-75 ^and 1e^-30 ^(between 8.8 and 9.4%), while it increased slowly up to 11.2% at a cut-off of 1e^-05^. The first chimerical contig was detected at a cut-off of 1e^-55 ^and up to 2.9 chimerical contigs for 10 Mb were observed at a cut-off of 1e^-05^. Thus, with SNaPshot data, cut-offs below 1e^-20 ^also lead to an increased number of chimerical contigs and therefore reduce assembly quality. The map length was estimated at each cut-off (Figure 2C) using a CB unit value of 1.1 kb ± 0.3 (see material and methods). The results show that at 1e^-75^, FPC builds a large number of very small contigs that results in a higher map length (266 Mb) than with WGP (Figure 2C). Then, the length decreases until 233 Mb (1e^-45^) and remains stable until 1e^-15^. Below this cut-off, the map length increases again due to an increased number of contigs (Figure 2A). The best possible final cut-off value to obtain a physical map optimized for the four criteria described above for the WGP was 1e^-25^.

To compare the performance and robustness of the two technologies for physical mapping in wheat, we used the results of the best physical maps obtained with each of them i.e. at a final cut-off of 1e^-25 ^for the SNaPshot and at a final cut-off of 1e^-11 ^for the WGP (Table 3). The results showed a higher proportion of singletons in WGP (36.9%) compared to the SNaPshot (18.8%) (Table 3). This is due to a large difference in the average number of tags per BAC obtained with WGP (17.3) vs. the number of bands per BAC obtained with the SNaPshot (117). As a result, the probability that there is enough information to merge two overlapping clones in a contig at an initial high stringency of 1e -75 is lower with the WGP technology than with the SNAPshot. Interestingly, the higher number of singletons did not affect greatly the size of the WGP assembly and the length of the WGP map (199 Mb ± 42) did not significantly differ from the length of the SNaPshot map (236 Mb ± 65). This was further confirmed by the results obtained for the 12 reference sequenced contigs (17.0 Mb for the WGP map vs. 17.2 Mb for the SNaPshot map). In fact, most of the singletons obtained with the WGP physical assembly correspond to BACs that are found in the depth rather than in the length of the map. Our results also indicated that WGP resulted in 31.2% fewer contigs than SNaPshot for an equivalent map length and a similar number of chimerical contigs (Table 3). The distribution of the contig size showed that in fact, the SNaPshot map contains many more small contigs (23.6% of contigs < 200 kb) than the WGP map (12.4% of contigs < 200 kb; Additional file 1). Finally, the comparison demonstrated that the WGP contigs contained 3.5 times fewer mis-assembled BACs than those obtained with the SNaPshot. Thus, we conclude that with less, but more accurate information Whole Genome Profiling builds more robust physical maps in wheat than the SNaPshot technology.

**Table 3:** Comparison of physical map assemblies obtained with the SNaPshot and WGP technologies at their optimum final cut-offs (1e-25 for SNaPshot; 1e-11 for WGP).

	SNaPshot	WGP
Total number of contigs	631	434
Average contigs size (Kb)	374^a^	469^b^
Median contigs size (Kb)	295^a^	374^b^
Number of singletons	2112 (18.8%)	4145 (36.9%)
Coverage in length	236 Mb ± 65^a^	199 Mb ± 42^b^

**Comparison with 12 reference sequenced contigs**		

Number of chimerical contigs for 10 Mb	0.6	0.6
Percentage of mis-assembled BACs	9.5%	2.7%

^b ^Based on an average bands size of 6.1 kb ± 1.3.

^a ^Based on an average bands size of 1,1 kb ± 0.3.

### Evaluating the potential for WGP to support the wheat genome sequencing effort

In addition to physical mapping, WGP provides sequence information assigned to individual BACs that may facilitate wheat genome sequence assembly by merging sequence contigs or scaffolds using the relative tag order. To evaluate this possibility, we tested the effect of integration of the WGP tags with 454 reads generated for four of the twelve reference BAC contigs from chromosome 3B [28]. The four contigs were resequenced in pools comprising four to nine BACs per pool, generating a total of 3,099,952 bp of sequence data, including 724,513 paired-end reads with an average length of 328 bp that provided 77-fold coverage of the reference contigs (Table 4). Random subsets of the sequences for the four pools were assembled to obtain eight different levels of coverage (15X to 50X), using paired-end and unpaired reads as well as with or without the integration of the WGP tags. Depending on the type of data used, three possible assembly outcomes were obtained: 1) assemblies performed with unpaired reads resulted in sequence contigs, 2) assemblies performed with paired-end reads without WGP tags integration as well as with unpaired reads integrated with the WGP tags resulted in scaffolds, and 3) assemblies performed with paired-end reads integrated with the WGP tags resulted in superscaffolds (SSCs). The N90 value was estimated as the length of the shortest contig (scaffold or superscaffold) such that the sum of contigs (scaffold or superscaffold) of equal length or longer covers at least 90% of the length of the 3.1 Mb of the 4 reference contigs. The L90 value, *i.e*. the minimum number of contigs or scaffolds or superscaffolds necessary to cover 90% of the 3.1 Mb of the four reference contigs, was also estimated and then both parameters were used to assess the quality of the assemblies. In wheat, sequence assemblies can be considered of high quality when the N90 is > 30 kb which is the size of the biggest transposable elements [28] and, with the smallest possible L90 value.

**Table 4:** Features of the 4 pools sequenced with the 454 GS-FLX technology.

	Number of BACs	Length of the reference sequence^a^	Number of reads^a^	Average reads size	Sequencing coverage
Pool1	9	1,135,279	241,916	323.0	69X
Pool2	6	665,389	157,653	334.7	79X
Pool3	4	622,598	151,278	328.2	80X
Pool4	5	676,686	173,666	328.6	84X

**TOTAL**	**24**	**3,099,952**	**724,513**	**328.0**	**77X**

^a ^The length of the sequences and reads are in bp.

**Figure 3:**
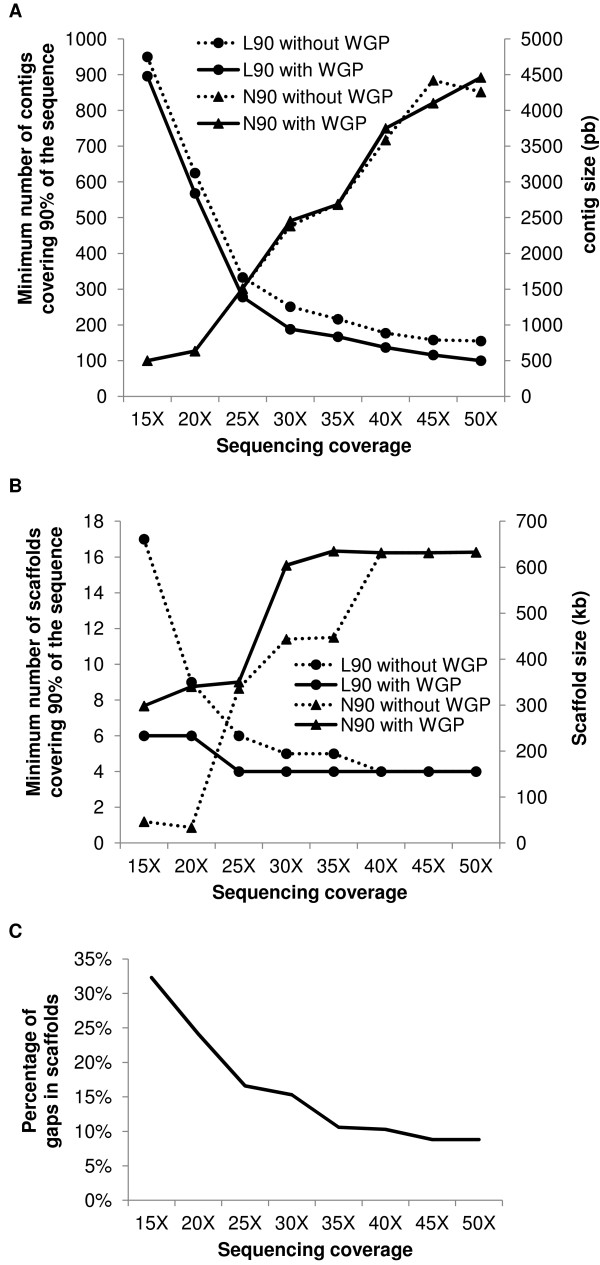
**Features of different sequence assemblies performed for four wheat BAC contigs representing 3,099,952 bp**. A) N90 and L90 values for different assemblies obtained with 454 sequencing with no paired-end reads as well as with (black line) and without (dotted line) the integration of WGP data between 15X and 50X sequencing coverage. B) N90 and L90 values for different assemblies obtained with 454 sequencing of paired-end reads and with and without integration of WGP data between 15X and 50X sequencing coverage. C) Percentage of gap in the scaffolds of different assemblies obtained with 454 paired-end reads between 15X and 50X sequencing coverage.

**Figure 4:**
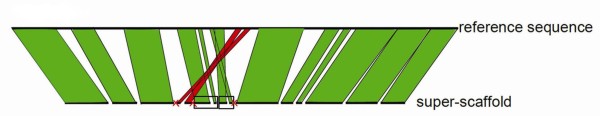
**Sequence alignment between a superscaffold obtained after WGP tags integration and a reference sequence (ctg0079)**. The superscaffold was obtained after assembly of 454 unpaired reads of ctg0079 with WGP tags integration at 40X coverage. Each line of the superscaffold corresponds to a contig and each rectangle corresponds to ambiguous contigs orders. Contigs within a bin are not ordered. Red areas highlight errors in contigs and bin mergers in the superscaffolds compared to the reference sequence. In this example, there are 12 mergers of which 3 are erroneous.

Assemblies with unpaired reads produced between 329 contigs at 50X coverage (Max contig size of 105 kb) and 950 contigs at 15X coverage (Max contig size of 28 kb; Additional file 2). The best assembly was obtained at a coverage of 50X and it had an N90 value of 4,259 bp and an L90 value of 155 (Figure 3A). Lower sequencing coverage led to decreased N90 and increased L90 values and thus a lower assembly quality. Information about the positions of the WGP tags was then incorporated into the 454 sequence assemblies (see Materials and Methods) thereby permitting some sequence contigs to be linked into scaffolds. On average, six scaffolds containing 10 contigs were obtained with a maximum size of 477 kb (Additional file 2). In principle, the relative order of the contigs in the scaffolds can be estimated from the positions of BAC-specific tags ordered along the WGP physical map. However, because the exact tag positions within a BAC are not known, the positions of some sequence contigs remained ambiguous (Figure 4) and, in 20% of the cases, the relative order proposed by the WGP integration was different from that in the reference sequence. Moreover, gaps represented 30% to 74.6% of regions in the assembled scaffolds (Additional file 3), likely resulting from tags with too low density in the WGP physical maps in combination with small sequence contig sizes. Here, it is important to bear in mind here that we were able to measure the gap sizes by comparing scaffolds with the available reference sequence; whereas, in a *de novo *analysis, the estimation of the gap sizes in the scaffolds would be impossible. At 40X sequencing coverage, the integration of WGP tags allowed us to obtain a quality, in terms of N90 and L90, equivalent to the 50X assembly without tag integration (N90 = 3.7 kb with the tag integration vs. 4.2 kb without the tags integration and L90 = 137 with the tag integration vs. 155 without the tag integration, respectively) (Figure 3A). We conclude that the integration of WGP tags in assemblies performed with unpaired reads enables the construction of scaffolds that could not be obtained otherwise even though these scaffolds will contain ordering errors and will lack a significant proportion of sequence information. Moreover, even at high sequencing coverage, the N90 value remained below the ~30 kb requested for reliable sequence assembly in wheat (Figure 3A). Although this type of sequence assembly cannot be considered as high quality, it can provide partial information at low cost.

The assemblies of the four BAC pools with 454 paired-end reads produced between 20 scaffolds at 45X coverage and 74 scaffolds at 15X coverage (Additional file 2). The best assemblies were obtained between 40X and 50X coverage and resulted in an N90 of ~632 kb and an L90 of 4 scaffolds that corresponds to the expected number of sequenced reference contigs (Figure 3B). At sequencing coverage between 25X and 35X, the L90 was of 5 or 6 scaffolds and the N90 ranged from 336 to 447 kb which still represents very high quality assemblies. At 15X and 20X coverage, the L90 value was 17 and 9 scaffolds respectively, and the N90 was 47 kb and 33.8 kb respectively, which remains acceptable. The scaffolds contained between 32.3% of gaps at 15X coverage and 8.8% at 50X coverage (Figure 3C). Integration of the WGP tags enabled to merge, on average, seven scaffolds into two SSCs (Additional file 2). No errors in the relative order of scaffolds in the SSCs were observed at 50X to 20X coverage levels; whereas, 20% of error was observed at 15X. The maximum percentage of gaps in the SSCs (*i.e*. gaps between scaffolds) was 3% at this coverage. Moreover, the WGP tag integration greatly improved the scaffolding at 15X and 20X by decreasing the L90 from 17 scaffolds to 6 SSCs and from 9 scaffolds to 6 SSCs, respectively, whereas the N90 value was increased from 46 to 298 kb and from 34 to 340 kb, respectively. Thus, we conclude that with paired-end sequence information, the integration of WGP tags can improve assemblies performed with low sequencing coverage (15X and 20X) but will result in assemblies containing more than 24% of gaps. At a coverage of 25X or higher, the integration of WGP tags does not significantly improve the quality of an assembly that is already high (N90 > 30kb, L90 close to the minimum and a percentage of gaps in scaffolds ≤ 20%) (Figure 3B).

In all, our results show that, in wheat, the integration of WGP sequence tags can help improve sequence scaffolding of pooled BACs, particularly at low sequence coverage; however, it does not reduce significantly the sequencing coverage necessary to maintain a high quality assembly in this complex genome.

## Discussion

### WGP enables the construction of robust physical maps in wheat

With current sequencing technologies, high quality draft sequences of genomes that contain a high level of TEs, such as maize [29] and wheat [6], can only be achieved by a clone-by-clone approach. With a size of 17 Gb, assembling a high quality reference sequence of the bread wheat genome remains costly even with NGS technologies. It is therefore essential that the minimal tiling path used for sequencing originates from a robust and accurate physical map. Because Whole Genome Profiling is based on the assembly of identical sequence tags, it is potentially more robust than any of the previous techniques used for physical mapping in wheat (e.g. SNaPshot [10,12]). Our results on a subset of about 1/3 of the largest wheat chromosome (3B, 1 Gb) demonstrate that, combined with an adapted assembly methodology, WGP offers a promising approach to construct robust and accurate physical maps of the wheat genome. The assembly methodology developed in this study enabled the construction of a physical map of an equivalent size to the one obtained with the SNaPshot technology [23] but with 30% fewer contigs and 3.5 times less mis-assembled BACs. The stepwise stringent assembly methodology produced more robust and accurate results for the large and complex genome of wheat than the single step methodology that was originally employed for developing WGP on *Arabidopsis thaliana *[26]. This implies that the WGP method can be adapted to any species by adjusting the assembly methodology and parameters.

The quality of a physical map assembly depends on the availability of sufficient information to establish contigs as well as the capacity to minimize the number of chimerical contigs. BAC library coverage and the density of bands/tags per BAC used to assemble contigs are the key factors for ensuring that adequate information is available. To date, contig assembly is done with the FPC software [27] that relies on the Sulston score [20] which corresponds to the probability of coincidence, *i.e*. the probability that two non-overlapping clones share by chance a given number of bands [27] or sequence tags in the case of WGP. The critical parameters are the selected tolerance to consider two bands/tags as identical and the cut-off value. The latter corresponds to the threshold of the Sulston score at which one considers that two clones do overlap. At a given cut-off, the higher the number of bands/tags per clone, the lower the percentage of overlap needed to merge two clones [7,21]. Thus, in WGP, the tag density affects directly the capacity to generate long contigs and, therefore, increasing the tag density should enhance the capacity to merge contigs, decrease the contig numbers, and consequently increase the physical map quality. In the present work, 327,282 tags were deconvoluted before tag and BAC filtering thereby resulting in a density of 23 tags per BAC, approximately 2.5 times less than the value theoretically expected if all tags could be deconvoluted (i.e. reflecting the *Eco*RI restriction enzyme recognition site frequency in the wheat genome). By comparison, in *Arabidopsis thaliana *the density was 40 tags per BAC and for melon, tomato, *Brassica napus*, and lettuce the tag densities were 26, 33, 22, and 25 [26]. Thus, the lower tag density observed in wheat than in *Arabidopsis *(and to a lesser extent tomato) may reduce the ability to exploit fully the advantages of WGP, even though this density is comparable to those obtained in melon, *Brassica napus*, and lettuce following digestion with *Eco*RI and *Mse*I. Thus, although the generated WGP map is of high quality, we suggest three possible ways to further improve quality metrics and reduce tag loss in WGP. The first improvement is directed at limiting the loss of information at the deconvolution steps through optimized design of the pooling strategy. The defined pooling strategy is a trade-off between the costs of sample preparation and sequencing using the Illumina GAII technology on the one hand and the size of the region covered by the BAC library (genome complexity) on the other [26]. The percentage of the genome covered in each pooling set has an impact on the tag loss at the deconvolution step. Indeed, at high coverage of the genome "in deconvolution space", there is a high probability that two or more BACs originating from the same region are present in the same pooling set. The WGP tags shared by these BACs will be lost at the deconvolution process as they are present in four or more BAC pools in a 2-D pooling scheme and six or more pools in a 3-D pooling scheme. In this study, the pooling strategy consisted of pooling sets of individual plates that each covers about 23% of the target region with a 3-D pooling scheme. If we had used the whole wheat chromosome 3B BAC library (56,952 clones) with the same 3-D pooling scheme, it is likely that fewer tags would have been lost as pooling would have covered only 5.3% of the total BAC library. Thus, our selection of BACs based on prior information likely decreased the "effective genome size" and thereby increased the loss of tags by deconvolution. Wheat chromosome sizes range from 600 Mb to 1Gb [30] while chromosome arms, currently used to construct the wheat physical maps in the framework of the IWGSC http://www.wheatgenome.org, range from 230 Mb to 580 Mb in size. Thus, if WGP is applied with a 3-D pooling scheme to the wheat chromosome arms, the pooling sets will cover between 9% and 23% of the genome/chromosome arm size. For the larger chromosome arms, less tags are expected to be lost at the deconvolution step. Another method for reducing tag loss due to a high coverage percentage of the pooling set would be to increase to 4, 5, or 6 the number of dimensions of the pooling set. However, this would significantly increase the cost of the experiment and the amount of sequence information needed which scales linearly with the number of dimensions (*i.e*. a 4-D scheme requires twice as much sequence information as a 2-D scheme). Thus, prior to each WGP project, the most cost efficient pooling scheme needs to be determined on the basis of the effective genome size.

Another improvement is to reduce the loss of information due to a too high number of identical tags identified in a pooling set by increasing the length of sequence reads. In *Arabidopsis*, the percentage of genome coverage for the pooling set was higher (40%) than in this study (23%) yet fewer tags were lost at the deconvolution step in *Arabidopsis *than in wheat. This suggests that a higher percentage of identical tags originating from different regions were found in the same pooling sets in wheat compared to *Arabidospis*, thereby limiting the probability of deconvoluting them, and, as a consequence, reducing the number of unique tags per BAC. This hypothesis is supported by the fact that the observed average number of BACs sharing the same tags was 2.9 instead of the expected 9.6 (*i.e*. BAC library coverage of the genome). The likely explanation for this observation is that since the wheat genome is many times (~120 fold) larger than *Arabidopsis *and consists of a large amount of repeated sequences, the tag length may not be optimal to avoid these confounding effects. Van Oeveren *et al *[26] with a simulation on the maize genome indicated that tag lengths of 26 to 31 nt should be sufficient for WGP, even for large genomes. For wheat, we suggest that 30 nt is sufficient to build robust physical maps but is not optimal to fully exploit WGP. To evaluate the potential of longer reads to decrease tag loss, we calculated an index of k-mer frequencies [31] on a 1X coverage sequence of the wheat genome http://www.cerealsdb.uk.net with k-mer sizes between 15 and 70 nt (data not shown). This showed that 71.1% of the 30-mers (corresponding to the length of a tag in this study) are unique but that increasing the tag length to 70 nt would improve the tag uniqueness up to 81.3%. Thus, the current improvement in read length and quality of the NGS technologies will likely provide opportunities for minimizing further tag loss and therefore improve the potential of WGP in wheat in the near future.

Finally, a third possibility for increasing tag density would be to choose an endonuclease that recognizes more abundant restriction sites in the wheat genome. The *Eco*RI enzyme used in this study shows a frequency of 1 site every 4.7 kb in wheat (based on the analysis of the reference sequence set of 18 Mb [28]). We recently observed in the same dataset that *Hin*dIII, also a 6 bp-cutter enzyme, shows a site frequency of 1 every 2.5 kb with the difference mainly caused by the composition of TE fraction (unpublished data). Selection of alternative restriction enzymes may therefore also be used to fine tune the performance of WGP in wheat.

The second important parameter for ensuring a high quality physical map is to limit as much as possible the number of chimerical contigs. Here, we estimated that, at the final cut-off value of 1e^-11 ^a number of chimerical contigs is 0.6 for 10 Mb of sequence. Van Oeveren *et al. *[26] have developed a methodology and a tool to identify chimerical contigs on the basis of the fraction of BAC pairs within a contig sharing at least one tag (C1) and the average tag density in a contig (C2). The authors empirically determined a threshold for which the square of C1 divided by C2 provided a value that discriminated between chimerical and non-chimerical contigs. Problematic BACs, then, can be identified and discarded by iteratively removing each BAC of the contig and testing whether BAC removal will break up the contig (23). We tested this approach on the wheat chromosome 3B dataset but it did not detect any of the chimerical contigs identified by comparison with the reference sequences. Moreover, only two contigs were identified as chimerical in the whole dataset with this approach while 14 were present based on our estimation of the number of chimerical contigs in 10 Mb. The threshold used to choose chimerical and contiguous contigs was defined from the WGP experiment on *Arabidopsis *[26] and it is likely that new parameters need to be established for wheat reinforcing the idea that parameters in the WGP analysis need to be adapted to the complexity of the target genome. With our dataset, we did not have sufficient sequence information to estimate a robust threshold value for wheat. The access to the entire 3B sequence in the near future (C. Feuillet, pers. comm.) will help in this regard.

### WGP tag integration improves low quality sequence assemblies and supports pooling strategies for achieving high quality sequence drafts

In addition to providing a robust physical map, WGP holds the potential of facilitating sequence assembly in whole genome or chromosome shotgun sequencing approaches. To date, with the current sequencing technologies, a whole genome or whole chromosome shotgun approach cannot be used to produce a high quality draft sequence of the wheat genome. Here, we wanted to investigate whether WGP can be used to further empower the BAC-by-BAC approach adopted by the IWGSC to obtain the reference sequence of each of the 21 individual chromosomes from the cv. Chinese Spring. Specifically, we wanted to see if WGP can reduce the costs of sequencing by providing a more robust physical framework and generating additional data that can support BAC contig sequence assemblies. The first BAC contig sequencing results obtained on chromosome 3B [28] using the physical map established with the SNaPshot technology [23] suggested that about 10% of the BACs were mis-assembled (unpublished data) and this was confirmed in our study (estimated mis-assembled BACs in the SNaPshot map: 9.5%). In a BAC-by-BAC approach, the mis-assembled BACs identified at the sequence assembly step need to be replaced by other BACs thereby increasing sequencing cost. By providing a physical map with less than 3% of mis-assembled BACs (2.7%), WGP thus would decrease the cost of sequencing by ~7% compared to a physical map constructed with the SNaPshot approach.

The assembly simulations indicated that with a low coverage/low cost sequencing approach (*i.e*. not based on the production of paired-end or mate-pair libraries) sequence assemblies can be improved significantly by integrating sequence tags produced by WGP. These assemblies contain, however, a significant amount of ambiguities in the contig order and a lack of knowledge about the percentage and distribution of gaps (at 25X and lower coverage, gaps represented about 50% of the assembled scaffolds). While such sequence cannot be considered a reference sequence, it can be used to develop molecular markers and perform preliminary comparative analysis. Assemblies with paired-end reads produce scaffolds for which the percentage and distribution of gaps can be estimated. At high sequence coverage (≥ 25 fold), such paired-end reads produce reliable assemblies whose quality cannot be significantly improved by integrating with WGP tags at the density produced in this study. In contrast, at low sequencing coverage (15X and 20X), WGP tag integration improves the assemblies by facilitating the construction of long, superscaffolds. However, such superscaffolds can include up to 15% of mis-ordered scaffolds that contain gaps of up to 30%. This type of sequence is comparable to the quality of sequence obtained at high coverage levels with unpaired reads. Thus, the complexity of the wheat genome with the presence of large and numerous transposable elements with highly similar sequences makes it impossible to produce a high quality reference sequence (≤ 20% of gaps in the sequence scaffolds and N90 > 30 kb) without any paired-end information and a minimum coverage of 25X. Thus, currently, WGP sequence tags produced during physical mapping are helpful to link sequence scaffolds but they cannot be used to decrease the sequencing coverage necessary to obtain a high quality assembly in such a complex genome.

In our opinion, the greatest potential for WGP is the possibility of increasing the degree of pooling in sequencing projects based on BAC pools, a strategy that has been proposed to reduce sequencing costs in large genomes [32] and is currently being utilized for sequencing chromosome 3B of bread wheat. In this case, the assembly of sequence reads from BAC pools of two or more unrelated physical contigs leads to sequence contigs (without paired-end) or scaffolds (with paired-end) that need to be reassigned to their respective physical contig of origin. WGP can provide the information needed for this assignment. In the 3B project, 52% of the 924 pools of the minimal tiling path used for 454 GS FLX Titanium sequencing contain two or more physical contigs (unpublished data). The 327,282 WGP tags generated in this pilot study will be helpful for assigning and ordering the sequence contigs or scaffolds produced from these pools.

## Conclusions

In this study, we evaluated the potential of WGP for physical mapping and sequencing of complex genomes like wheat. The comparison of wheat WGP and SNaPshot physical maps showed that, with an adapted assembly methodology, WGP needed 30% less contigs to cover the same regions than the SNaPshot physical map and that WGP contigs contained 3.5 times less mis-assembled BACs. Thus, we conclude that, with a suitable assembly methodology, WGP builds more robust physical maps than the SNaPshot technology in wheat and that WGP can be adapted to any genome. Moreover, we evaluated the benefit of integrating WGP tags in different sequence assemblies obtained after Next Generation Sequencing of BAC pools. The results showed that WGP tag integration improves low quality sequence assembly. However, to achieve a high quality draft sequence assembly, a sequencing depth of 25x Roche/454 paired-end reads is required, at which point WGP tag integration does not provide additional scaffolding value. Nonetheless, WGP tags will support the efficient sequencing of BAC pools by enabling reliable assignment of sequence scaffolds to their BAC of origin.

## Methods

### BAC library and SNaPshot fingerprinting

A subset of 16,128 BAC clones from the 3B BAC library that comprises a total of 56,952 BAC clones (representing 9.6x coverage of wheat chromosome 3B) was used to assemble a physical map with the WGP and SNaPshot technologies. Selection criteria of the subset are described in the results section and details on the BAC library construction, SNaPshot fingerprinting reaction, and data processing are described by Paux *et al *[23].

### WGP data production

The WGP data production process was performed essentially as described by Van Oeveren *et al *[26] and encompassed the following steps: a) Pooling individual BAC clones in a 3-dimensional (3-D) format, comprising pools with two rows (48 BACs each), pools with three columns (48 BACs each) and an additional set of pools to differentiate the group of 6 BACs labeled by a single row and column pool combination (termed split-box pools with 64 BACs each). This results for each 384-well plate of BAC clones in 8 row pools, 8 column pools and 6 split-box pools and allows four 384-well plates to fill 88 pooled wells in a single 96-wells plate; b) Isolation of high concentration, low *E. coli *level, pooled BAC DNA (Amplicon Express); c) Digestion with the *Eco*RI/*Mse*I restriction enzymes, ligation of Illumina GA adaptor sequences containing sample identification tags (barcodes) and PCR amplification; d) Pooling of the PCR products; e) Cluster amplification; and f) Sequencing using the GAII with 36 nt read length. GAII sequencing resulted in a total of 96.3 million high-quality sequence reads. These reads were used for WGP data processing, which included the following steps: a) Identification of barcodes and restriction site and linking sequence reads to BAC pool IDs; b) Deconvolution, *i.e*. assignation of sequence reads as WGP tags to individual BACs from the 3-D pool information; and c) Filtering of the WGP tags using various quality control measures for further noise reduction.

### Tag distribution and sequenced contigs

Twelve reference contigs ctg0005, ctg0011, ctg0079, ctg0091, ctg0382, ctg0464, ctg528, ctg0616, ctg0661, ctg0954, ctg1030 and ctg1035 that were Sanger sequenced and manually annotated previously were used to control the robustness of the WGP physical map. BAC sequencing, assembly, and annotation are described in [28]. All tags belonging to BACs from these 12 contigs were mapped on the 12 sequences by BLASTn [33] (100% identity and 100% tag coverage). For complete information on these BACs, see [23] and [28].

### Physical map construction

Except for the first WGP assembly, all physical map assemblies were performed using the methodology described by Paux *et al *[23] for the construction of the physical map of chromosome 3B. Briefly, the initial build was performed by incremental contig building with a cut-off of 1e^-75^. These were subsequently run through single-to-end and end-to-end merging (Match: 1) at 15 successively higher cut-offs ending at 1e^-05^. The DQer function was used at each cut-off to break up all contigs that contained more than 10% of Questionable (Q) clones (Step: 3).

The following parameters were used to establish the physical map with the fingerprints obtained by the SNaPshot technology: a gel length of 18,000, a FromEnd value of 55 and a tolerance of 4 [23]. For the WGP technology, the parameters were: a gel length of 110,000, a FromEnd of 8 and a tolerance of 0, except for the first assembly that was performed with the methodology and parameters described by Van Oeveren et al. [26]: a gel length of 3,300 (default parameter), a single cut-off and DQing step at e-06, a FromEnd of 15 and a tolerance of 0.

### Percentage of chimerical contigs and mis-assembled BACs estimation

The BAC dataset comprised of 1,380 BACs belonging to 12 reference sequenced contigs was used as control to assess the accuracy of the WGP assemblies. A total of 3,396 WGP tags were mapped on these 12 sequenced contigs (full-length alignment and 100% identity). All BACs with more than 50% tags mapped were considered as "matched BACs". All contigs with at least two overlapping matched BACs were manually checked. All contigs covering non-contigous regions were considered as chimerical contigs. The percentage of mis-assembled BACs was estimated in the non-chimerical contigs and in the regions matching the sequences of chimerical contigs on the basis of BACs with less than 50% of their tags mapped in the reference sequences.

### Estimation of a CB unit size

We determined the average size of a CB unit (the unit of the length of contigs in FPC, *i.e*. the average distance between bands or tags in the contig) for the WGP and SNaPshot physical contigs by mapping the contigs with the sequence tags obtained on the 12 reference sequenced contigs and calculating the ratio between the size in kb and the size in CB unit for each contig.

### Roche 454 contig sequencing and assembly

Four BAC pools corresponding to four physical contigs were sequenced on a Roche/454 GS FLX sequencer at the Centre National de Séquençage (Evry, France) in the framework of the project aiming at sequencing chromosome 3B (3BSEQ, http://urgi.versailles.inra.fr/Projects/3BSeq; unpublished data). Mate pair libraries of 8 kb were generated for pools 1, 2, 3, and 4 that contained 9 BACs of ctg0005, 6 BACs of ctg0079, 4 BACs of ctg0382, and 5 BACs of ctg0091, respectively. These 4 contigs are part of the 12 reference contigs previously sequenced by Sanger Sequencing and annotated [28]. Pools 1 to 4 were sequenced at a final coverage of 69X, 79X, 80X, and 84X, respectively. The list of the sequenced BACs and the size of the sequence covered by the pools are shown in the Additional file 4.

To simulate the effect of the sequencing depth/coverage on the quality of the assembly, each set of 454 reads from each BAC pool was divided into subsets. The 454 reads were randomly selected to obtain subsets with coverage of 50X, 45X, 40X, 35X, 30X, 25X, 20X, and 15X and each subset was assembled using the gsAssembler v2.3 (Roche). The resulting contigs were considered as obtained from unpaired reads whereas the scaffolds were considered as obtained from paired-end reads in the subsequent analyses. These contigs and scaffolds were used to evaluate the impact of the integration of WGP tags on sequence scaffolding.

### Integration of the WGP tags into sequence assemblies

The 47,220 WGP tags were integrated into the different Roche 454 sequence assemblies (see above) for the four contigs using the following criteria: a minimum match with 100% identity of at least two different tags from two distinct restriction sites on a single WGP physical contig or singleton was required to permit the link with a sequence contig. Both links between single WGP contigs and multiple sequence contigs and links between multiple WGP contigs and single sequence contigs can occur. The sequence contigs with a match to a single WGP contig were linked to create a sequence scaffold based on the average matched position. For large sequence contigs that matched multiple WGP contigs, the WGP contigs were ordered according to the tag positions on the sequence. This process led to the construction of superscaffolds (SSC) and was repeated until no further links or only conflicting links were found.

## Authors' contributions

RP performed data analysis, interpretation of results and drafted the manuscript. FC and EP participated in designing the study and interpreting the results. JvO participated in the design of the WGP study and performed the WGP analysis. JT and AJ developed the tool for integration of the WGP and the sequence data sets. AW, MvE, and KS participated in the concept and design of the WGP study. AA, PW and EA performed the sequencing experiments. EvdV participated in the design and the coordination of the WGP study. CF participated in the design of the study, interpretation of the results and revised the manuscript. All authors have read and approved the final manuscript.

## Supplementary Material

Additional file 1 **Distribution of the contig size in the optimal WGP and SNaPshot physical maps**. Click here for file

Additional file 2 **Summary of the assemblies of 454 reads from 24 BACs representing 3,099,952 bp performed with or without Paired-End reads and with or without the integration of WGP tags at different sequencing coverage**. Click here for file

Additional file 3 **Percentage of gaps in the scaffolds obtained by integrating WGP tags with 454 unpaired reads at different levels of reference contig coverage**. Click here for file

Additional file 4 **List of the BACs sequenced in each pool and size of the sequence covered by each pool**. Click here for file

## References

[B1] Collins F Has the revolution arrived? Nature 2010 464 7289 674 675 10.1038/464674a 20360716 PMC5101928

[B2] Feuillet C Leach JE Rogers J Schnable PS Eversole K Crop genome sequencing: lessons and rationales Trends in Plant Science 2011 16 2 77 88 10.1016/j.tplants.2010.10.005 21081278

[B3] Chain PS Grafham DV Fulton RS Fitzgerald MG Hostetler J Muzny D Ali J Birren B Bruce DC Buhay C Genomics. Genome project standards in a new era of sequencing Science 2009 326 5950 236 237 10.1126/science.1180614 19815760 PMC3854948

[B4] Green ED Strategies for the systematic sequencing of complex genomes Nat Rev Genet 2001 2 8 573 583 10.1038/35084503 11483982

[B5] Miller JR Koren S Sutton G Assembly algorithms for next-generation sequencing data Genomics 2010 95 6 315 327 10.1016/j.ygeno.2010.03.001 20211242 PMC2874646

[B6] Smith DB Flavell RB Characterization of Wheat Genome by Renaturation Kinetics Chromosoma 1975 50 3 223 242

[B7] Ding Y Johnson MD Chen WQ Wong D Chen YJ Benson SC Lam JY Kim YM Shizuya H Five-color-based high-information-content fingerprinting of bacterial artificial chromosome clones using type IIS restriction endonucleases Genomics 2001 74 2 142 154 10.1006/geno.2001.6547 11386750

[B8] Green ED Green P Sequence-tagged site (STS) content mapping of human chromosomes: theoretical considerations and early experiences PCR Methods Appl 1991 1 2 77 90 1842934 10.1101/gr.1.2.77

[B9] Jiang J Gill BS Current status and the future of fluorescence in situ hybridization (FISH) in plant genome research Genome 2006 49 9 1057 1068 10.1139/g06-076 17110986

[B10] Luo MC Thomas C You FM Hsiao J Ouyang S Buell CR Malandro M McGuire PE Anderson OD Dvorak J High-throughput fingerprinting of bacterial artificial chromosomes using the snapshot labeling kit and sizing of restriction fragments by capillary electrophoresis Genomics 2003 82 3 378 389 10.1016/S0888-7543(03)00128-9 12906862

[B11] Marra M Kucaba T Sekhon M Hillier L Martienssen R Chinwalla A Crockett J Fedele J Grover H Gund C A map for sequence analysis of the *Arabidopsis thaliana *genome Nat Genet 1999 22 3 265 270 10.1038/10327 10391214

[B12] Nelson WM Dvorak J Luo MC Messing J Wing RA Soderlund C Efficacy of clone fingerprinting methodologies Genomics 2007 89 1 160 165 10.1016/j.ygeno.2006.08.008 17011744

[B13] Xu ZY Sun SK Covaleda L Ding K Zhang AM Wu CC Scheuring C Zhang HB Genome physical mapping with large-insert bacterial clones by fingerprint analysis: methodologies, source clone genome coverage, and contig map quality Genomics 2004 84 6 941 951 10.1016/j.ygeno.2004.08.014 15533711

[B14] Lin L Pierce GJ Bowers JE Estill JC Compton RO Rainville LK Kim C Lemke C Rong J Tang H A draft physical map of a D-genome cotton species (Gossypium raimondii) BMC Genomics 2010 11 10.1186/1471-2164-11-395 PMC2996926 20569427

[B15] Marra MA Kucaba TA Dietrich NL Green ED Brownstein B Wilson RK McDonald KM Hillier LW McPherson JD Waterston RH High throughput fingerprint analysis of large-insert clones Genome Research 1997 7 11 1072 1084 9371743 10.1101/gr.7.11.1072 PMC310686

[B16] Ragupathy R Rathinavelu R Cloutier S Physical mapping and BAC-end sequence analysis provide initial insights into the flax (Linum usitatissimum L.) genome BMC Genomics 2011 12 10.1186/1471-2164-12-217 PMC3113786 21554714

[B17] Zhang X Scheuring CF Zhang M Dong JJ Zhang Y Huang JJ Lee M-K Abbo S Sherman A Shtienberg D A BAC/BIBAC-based physical map of chickpea, Cicer arietinum L BMC Genomics 2010 11 501 20849583 10.1186/1471-2164-11-501 PMC2996997

[B18] McPherson JD Marra M Hillier L Waterston RH Chinwalla A Wallis J Sekhon M Wylie K Mardis ER Wilson RK A physical map of the human genome Nature 2001 409 6822 934 941 10.1038/35057157 11237014

[B19] Chen M Presting G Barbazuk WB Goicoechea JL Blackmon B Fang G Kim H Frisch D Yu Y Sun S An integrated physical and genetic map of the rice genome Plant Cell 2002 14 3 537 545 10.1105/tpc.010485 11910002 PMC150577

[B20] Soderlund C Humphray S Dunham A French L Contigs built with fingerprints, markers, and FPC V4.7 Genome Res 2000 10 11 1772 1787 10.1101/gr.GR-1375R 11076862 PMC310962

[B21] Ding Y Johnson MD Colayco R Chen YJ Melnyk J Schmitt H Shizuya H Contig assembly of bacterial artificial chromosome clones through multiplexed fluorescence-labeled fingerprinting Genomics 1999 56 3 237 246 10.1006/geno.1998.5734 10087190

[B22] Zhang HB Wing RA Physical mapping of the rice genome with BACs Plant Molecular Biology 1997 35 1-2 115 127 9291965

[B23] Paux E Sourdille P Salse J Saintenac C Choulet F Leroy P Korol A Michalak M Kianian S Spielmeyer W A Physical Map of the 1-Gigabase Bread Wheat Chromosome 3B Science 2008 322 5898 101 104 10.1126/science.1161847 18832645

[B24] Wei F Coe E Nelson W Bharti AK Engler F Butler E Kim H Goicoechea JL Chen M Lee S Physical and Genetic Structure of the Maize Genome Reflects Its Complex Evolutionary History PLoS Genet 2007 3 7 e123 10.1371/journal.pgen.0030123 17658954 PMC1934398

[B25] Nelson W Soderlund C Integrating sequence with FPC fingerprint maps Nucleic Acids Research 2009 37 5 10.1093/nar/gkp034 PMC2655663 19181701

[B26] van Oeveren J de Ruiter M Jesse T van der Poel H Tang JF Yalcin F Janssen A Volpin H Stormo KE Bogden R Sequence-based physical mapping of complex genomes by whole genome profiling Genome Research 2011 21 4 618 625 10.1101/gr.112094.110 21324881 PMC3065709

[B27] Soderlund C Longden I Mott R FPC: a system for building contigs from restriction fingerprinted clones Comput Appl Biosci 1997 13 5 523 535 9367125 10.1093/bioinformatics/13.5.523

[B28] Choulet F Wicker T Rustenholz C Paux E Salse J Leroy P Schlub S Le Paslier MC Magdelenat G Gonthier C Megabase level sequencing reveals contrasted organization and evolution patterns of the wheat gene and transposable element spaces Plant Cell 2010 22 6 1686 1701 10.1105/tpc.110.074187 20581307 PMC2910976

[B29] Schnable PS Ware D Fulton RS Stein JC Wei F Pasternak S Liang C Zhang J Fulton L Graves TA The B73 maize genome: complexity, diversity, and dynamics Science 2009 326 5956 1112 1115 10.1126/science.1178534 19965430

[B30] Dolezel J Simkova H Kubalakova M Safar J Suchankova P Cihalikova J Bartos J Valarik M Feuillet C, Muehlbauer GJ Chromosome Genomics in the Triticeae Plant Genetics and Genomics 2009 Genetics and Genomics of the Triticeae: Crops and Models 7 New York: Springer 285 316

[B31] Kurtz S Narechania A Stein JC Ware D A new method to compute K-mer frequencies and its application to annotate large repetitive plant genomes BMC Genomics 2008 9 517 10.1186/1471-2164-9-517 18976482 PMC2613927

[B32] Rounsley S Marri P Yu Y He R Sisneros N Goicoechea J Lee S Angelova A Kudrna D Luo M De Novo Next Generation Sequencing of Plant Genomes Rice 2009 2 1 35 43 10.1007/s12284-009-9025-z

[B33] Altschul SF Madden TL Schaffer AA Zhang JH Zhang Z Miller W Lipman DJ Gapped BLAST and PSI-BLAST: a new generation of protein database search programs Nucleic Acids Research 1997 25 17 3389 3402 10.1093/nar/25.17.3389 9254694 PMC146917

