# Supplementary figures and images for: Whole Genome Profiling provides a robust framework for physical mapping and sequencing in the highly complex and repetitive wheat genome
Source: BMC Genomics. 2012 Jan 30;13:47. doi: 10.1186/1471-2164-13-47 (PMC3311077; doi:10.1186/1471-2164-13-47)

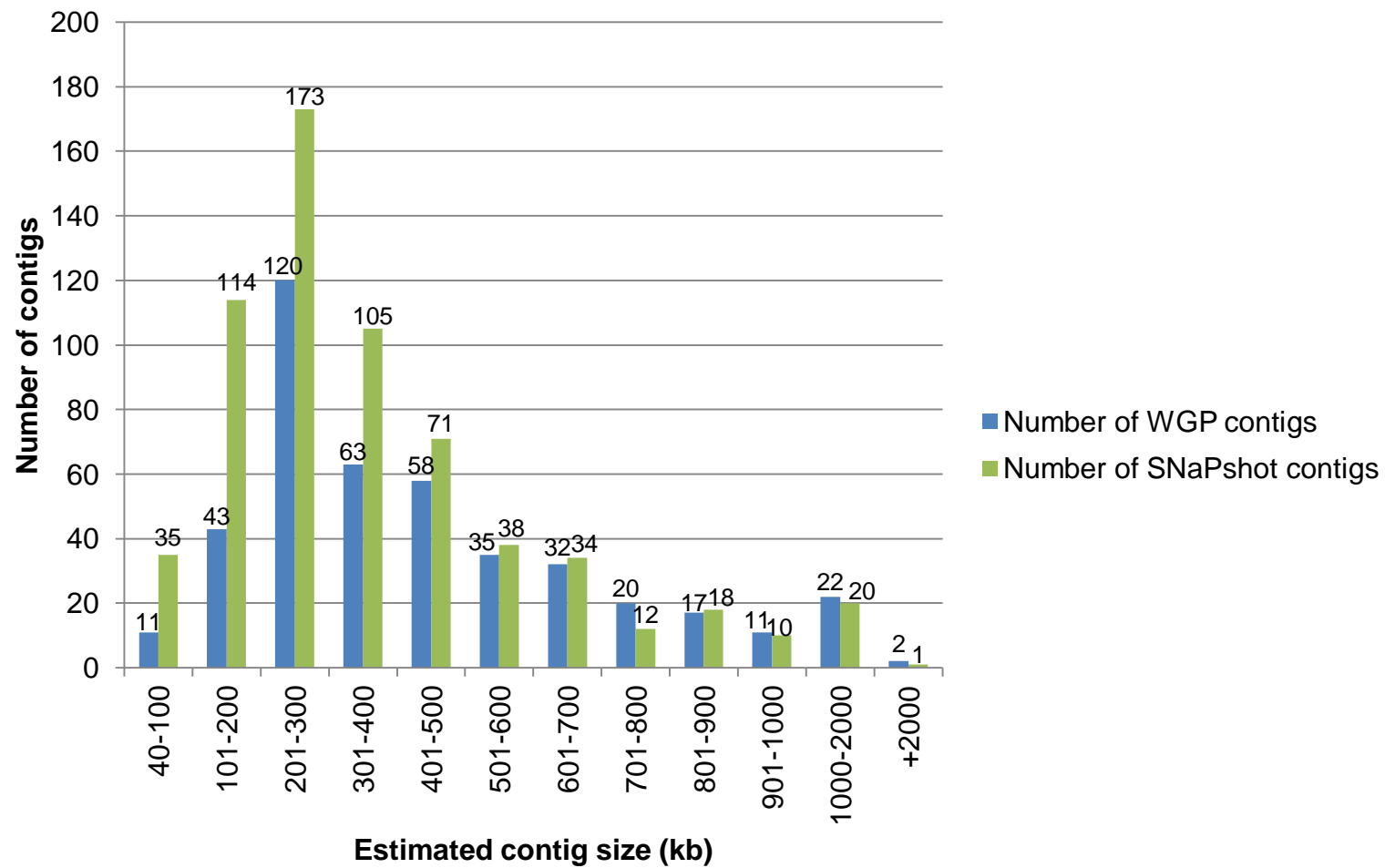

Additional file 1. Distribution of the contig size in the optimal WGP and SNaPshot physical maps.

Supplement: Additional file 1 — Distribution of the contig size in the optimal WGP and SNaPshot physical maps. [file 1471-2164-13-47-S1.PDF]
