# Supplementary material for: Whole Genome Profiling provides a robust framework for physical mapping and sequencing in the highly complex and repetitive wheat genome
Source: BMC Genomics. 2012 Jan 30;13:47. doi: 10.1186/1471-2164-13-47 (PMC3311077; doi:10.1186/1471-2164-13-47)
Supplement: Additional file 2 — Summary of the assemblies of 454 reads from 24 BACs representing 3,099,952 bp performed with or without Paired-End reads and with or without the integration of WGP tags at different sequencing coverage. [file 1471-2164-13-47-S2.PDF]

Additional file 2. Summary of the assemblies of 454 reads from 24 BACs representing 3,099,952 bp performed with or without Paired-end and with or without the integration of WGP tags at different sequencing coverage

	Without Paired-End assemblies											
	Without WGP tag integration						With WGP tag integration					
Sequencing coverage	Number of contigs	Median contig size	Max contigs size (bp)	Contigs total size (bp)	N90 (bp)	L90	Number of superscaffolds	Number of contigs in the superscaffolds	Max scaffolds size (bp)	Scaffolds total size (bp)	N90 (bp)	L90
**50X**	329	3649	105,367	3,052,310	4259	155	6	63	473,550	1,751,409	4461	100
**45X**	336	3628	116,518	3,040,086	4422	158	5	54	467,283	1,495,997	4103	116
**40X**	338	4618	90,775	3,019,501	3588	177	6	46	476,991	1,293,269	3750	137
**35X**	397	3437	91,556	3,003,724	2691	216	7	59	348,475	1,379,354	2683	167
**30X**	425	3818	78,326	2,989,018	2385	251	6	68	429,284	1,460,183	2453	188
**25X**	502	3169	108,984	2,949,267	1509	333	6	61	347,386	1,177,562	1509	278
**20X**	710	1903	56,099	2,838,673	634	625	7	64	237,641	948,264	634	568
**15X**	950	1411	28,174	2,659,233	500	950	5	60	223,826	623,501	500	896

	With Paired-End assemblies											
	Without WGP tag integration						With WGP tag integration					
Sequencing coverage	Number of scaffolds	Max scaffolds size (bp)	Scaffolds total size (bp)	Gap percentage in scaffolds (%)	N90 (kb)	L90	Number of superscaffolds	Number of scaffolds in the superscaffolds	Max superscaffolds size (bp)	Superscaffolds total size (bp)	N90 (kb)	L90
**50X**	26	1,174,455	3,224,519	8.8%	632.7	4	1	2	684,253	684,253	632.7	4
**45X**	20	1,192,731	3,217,546	8.8%	631.5	4	0	0	0	0	631.5	4
**40X**	28	1,137,139	3,228,479	10.3%	631.5	4	1	2	1,181,186	1,181,186	631.5	4
**35X**	22	730,142	3,200,223	10.6%	447.4	5	2	4	1,177,572	1,812,599	635.0	4
**30X**	38	725,531	3,246,400	15.3%	443.5	5	2	4	1,169,073	1,825,023	604.4	4
**25X**	35	729,543	3,299,026	16.6%	336.4	6	2	5	1,184,034	1,817,777	350.2	4
**20X**	53	617,536	3,299,138	24.1%	33.8	9	4	14	903,180	2,854,113	340.1	6
**15X**	74	566,582	3,229,635	32.3%	46.6	17	5	23	664,409	2,570,204	298.3	6
