# Supplementary material for: Whole Genome Profiling provides a robust framework for physical mapping and sequencing in the highly complex and repetitive wheat genome
Source: BMC Genomics. 2012 Jan 30;13:47. doi: 10.1186/1471-2164-13-47 (PMC3311077; doi:10.1186/1471-2164-13-47)
Supplement: Additional file 3 — Percentage of gaps in the scaffolds obtained by integrating WGP tags with 454 unpaired reads at different levels of reference contig coverage. [file 1471-2164-13-47-S3.PDF]

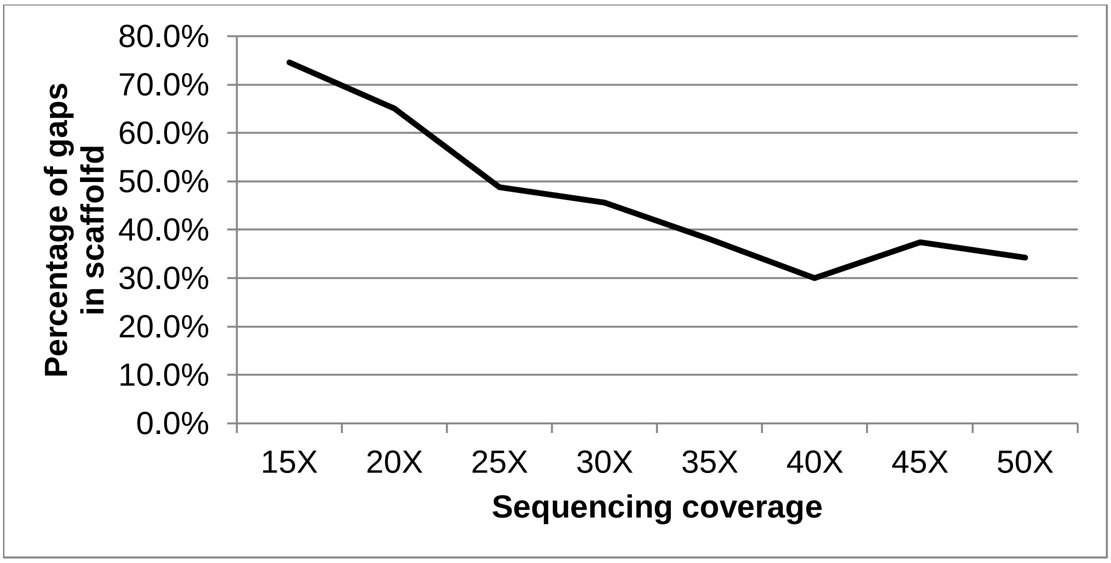

Additional file 3. Percentage of gaps in the scaffolds obtained by integrating WGP tags with 454 unpaired read data at differents levels of reference contig coverage
