# Supplementary material for: Whole Genome Profiling provides a robust framework for physical mapping and sequencing in the highly complex and repetitive wheat genome
Source: BMC Genomics. 2012 Jan 30;13:47. doi: 10.1186/1471-2164-13-47 (PMC3311077; doi:10.1186/1471-2164-13-47)
Supplement: Additional file 4 — List of the BACs sequenced in each pool and size of the sequence covered by each pool. [file 1471-2164-13-47-S4.PDF]

Additional file 4. List of the sequenced BACs in each pool and size of the sequence covered by these pools

Pool 1 covering 1,135,279 bp:

TaaCsp3BFhA_0040D16	ctg0005
TaaCsp3BFhA_0045N23	ctg0005
TaaCsp3BFhA_0115C24	ctg0005
TaaCsp3BFhA_0001C11	ctg0005
TaaCsp3BFhA_0109N19	ctg0005
TaaCsp3BFhA_0002C20	ctg0005
TaaCsp3BFhA_0034E06	ctg0005
TaaCsp3BFhA_0015P16	ctg0005
TaaCsp3BFhA_0068G05	ctg0005

Pool 2 covering 665,391 bp:

TaaCsp3BFhA_0149J15	ctg0079
TaaCsp3BFhA_0013G07	ctg0079
TaaCsp3BFhA_0061H24	ctg0079
TaaCsp3BFhA_0023N15	ctg0079
TaaCsp3BFhA_0072J16	ctg0079
TaaCsp3BFhA_0097H10	ctg0079

Pool 3 covering 622,598 bp:

TaaCsp3BFhA_0053M11	ctg0382
TaaCsp3BFhA_0089F10	ctg0382
TaaCsp3BFhA_0096J17	ctg0382
TaaCsp3BFhA_0108O13	ctg0382
TaaCsp3BFhA_0028O12	ctg0091
TaaCsp3BFhA_0035C19	ctg0091
TaaCsp3BFhA_0076E14	ctg0091
TaaCsp3BFhA_0140I02	ctg0091
TaaCsp3BFhA_0037M16	ctg0091

Pool 4 covering 676,686 bp:

TaaCsp3BFhA_0028O12	ctg0091
TaaCsp3BFhA_0035C19	ctg0091
TaaCsp3BFhA_0076E14	ctg0091
TaaCsp3BFhA_0140I02	ctg0091
TaaCsp3BFhA_0037M16	ctg0091
